# Tris(*o*-chloro­benz­yl)[3-(4-meth­oxybenzoyl)­propionato-κ*O*]tin(IV)

**DOI:** 10.1107/S1600536810005921

**Published:** 2010-02-20

**Authors:** Thy Chun Keng, Kong Mun Lo, Seik Weng Ng

**Affiliations:** a Department of Chemistry, University of Malaya, 50603 Kuala Lumpur, Malaysia

## Abstract

The tin atom in the title compound, [Sn(C_7_H_6_Cl)_3_(C_11_H_11_O_4_)], exists in a distorted tetra­hedral coordination environment. The carboxyl­ate anion is equally disordered over two positions.

## Related literature

Trialkyl­tin carboxyl­ates are generally carboxyl­ate-bridged polymers; see: Ng & Kumar Das (1991 ▶). For the direct synthesis of substituted tribenzyl­tin chlorides, see: Sisido *et al.* (1961 ▶).
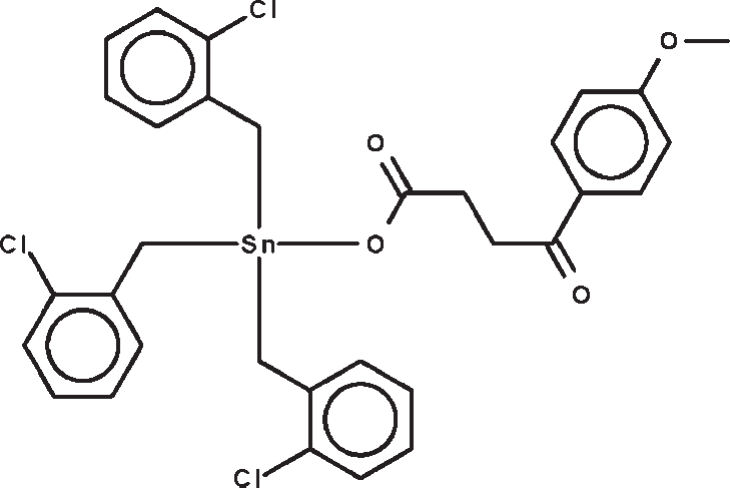


## Experimental

### 

#### Crystal data

[Sn(C_7_H_6_Cl)_3_(C_11_H_11_O_4_)]
*M* *_r_* = 702.59
Triclinic, 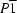

*a* = 8.4934 (6) Å
*b* = 9.2077 (7) Å
*c* = 21.3736 (16) Å
α = 82.592 (1)°
β = 85.654 (1)°
γ = 66.757 (1)°
*V* = 1522.47 (19) Å^3^
*Z* = 2
Mo *K*α radiation
μ = 1.14 mm^−1^
*T* = 293 K
0.30 × 0.20 × 0.10 mm

#### Data collection

Bruker SMART APEX diffractometer
Absorption correction: multi-scan (*SADABS*; Sheldrick, 1996 ▶) *T*_min_ = 0.795, *T*_max_ = 1.000
14685 measured reflections
6922 independent reflections
5062 reflections with *I* > 2σ(*I*)
*R*_int_ = 0.027

#### Refinement

*R*[*F* ^2^ > 2σ(*F* ^2^)] = 0.041
*wR*(*F* ^2^) = 0.114
*S* = 1.03
6922 reflections
348 parameters
116 restraints
H-atom parameters constrained
Δρ_max_ = 0.39 e Å^−3^
Δρ_min_ = −0.41 e Å^−3^

### 

Data collection: *APEX2* (Bruker, 2009 ▶); cell refinement: *SAINT* (Bruker, 2009 ▶); data reduction: *SAINT*; program(s) used to solve structure: *SHELXS97* (Sheldrick, 2008 ▶); program(s) used to refine structure: *SHELXL97* (Sheldrick, 2008 ▶); molecular graphics: *X-SEED* (Barbour, 2001 ▶); software used to prepare material for publication: *publCIF* (Westrip, 2010 ▶).

## Supplementary Material

Crystal structure: contains datablocks global, I. DOI: 10.1107/S1600536810005921/xu2728sup1.cif

Structure factors: contains datablocks I. DOI: 10.1107/S1600536810005921/xu2728Isup2.hkl

Additional supplementary materials:  crystallographic information; 3D view; checkCIF report

## Figures and Tables

**Fig. 1.**
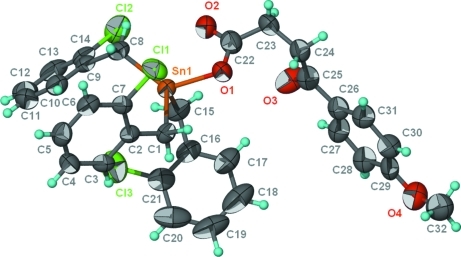
Thermal ellipsoid plot (Barbour, 2001) of Sn(C7H6Cl)3(C11H11O4) at the 50% probability level. Hydrogen atoms are drawn as spheres of arbitrary radius. The disorder component of the carboxylate anion is shown in the monodentate mode.

**Fig. 2.**
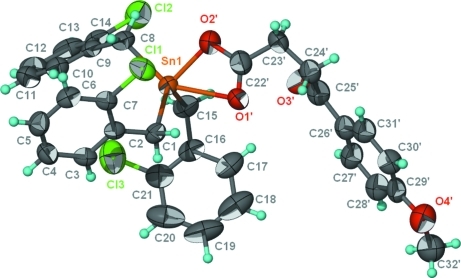
Thermal ellipsoid plot (Barbour, 2001) of Sn(C7H6Cl)3(C11H11O4) at the 50% probability level. Hydrogen atoms are drawn as spheres of arbitrary radius. The disorder component of the carboxylate anion is shown in the chelating mode.

## supplementary crystallographic information

### Experimental

3-(4-Methoxyl)benzoylpropionic acid (0.1 g, 0.5 mmol) and tri(*o*-chlorobenzyl)tin hydroxide (0.25 g, 0.5 mmol) were heated in ethanol (100 ml) for 1 h. After filtering of the mixture, colorless crystals were obtained upon slow evaporation of the filtrate.

### Refinement

Hydrogen atoms were placed at calculated positions (C–H 0.93–0.97 Å) and were treated as riding on their parent atoms, with *U*(H) set to 1.2–1.5 times *U*_eq_(C). The aromatic rings of the benzyl group were refined as rigid hexagons of 1.39 Å sides.

The carboxylate anion is disordered over two positions; the occupancy could not be refined, and was assumed to be a 1:1 type of disorder.

The C–O distances were restrained to 1.25±0.01 Å; the 1,2-related carbon-carbon distances in the chain were restrained to 1.50±0.01 Å and the 1,3-related ones to 2.35±0.01 Å. For the methoxy portion, the carbon-oxygen distances were restrained to 1.35±0.01 Å. The four-atom unit for the carbony/carboxyl portions were restrained to be nearly flat. The temperature factors of the primed atoms were restrained to those of the unprimed ones, and the anisotropic temperature factors of the disordered atoms were restrained to be nearly isotropic. The aromatic rings were treated as rigid hexagons.

### Crystal data

**Table:**

[Sn(C_7_H_6_Cl)_3_(C_11_H_11_O_4_)]	*Z* = 2
*M**_r_* = 702.59	*F*(000) = 708
Triclinic, *P*1	*D*_x_ = 1.533 Mg m^−^^3^
Hall symbol: -P 1	Mo *K*α radiation, λ = 0.71073 Å
*a* = 8.4934 (6) Å	Cell parameters from 3994 reflections
*b* = 9.2077 (7) Å	θ = 2.4–22.1°
*c* = 21.3736 (16) Å	µ = 1.14 mm^−^^1^
α = 82.592 (1)°	*T* = 293 K
β = 85.654 (1)°	Prism, colorless
γ = 66.757 (1)°	0.30 × 0.20 × 0.10 mm
*V* = 1522.47 (19) Å^3^	

### Data collection

**Table:**

Bruker SMART APEX diffractometer	6922 independent reflections
Radiation source: fine-focus sealed tube	5062 reflections with *I* > 2σ(*I*)
graphite	*R*_int_ = 0.027
ω scans	θ_max_ = 27.5°, θ_min_ = 1.0°
Absorption correction: multi-scan (*SADABS*; Sheldrick, 1996)	*h* = −11→10
*T*_min_ = 0.795, *T*_max_ = 1.000	*k* = −11→11
14685 measured reflections	*l* = −27→27

### Refinement

**Table:**

Refinement on *F*^2^	Primary atom site location: structure-invariant direct methods
Least-squares matrix: full	Secondary atom site location: difference Fourier map
*R*[*F*^2^ > 2σ(*F*^2^)] = 0.041	Hydrogen site location: inferred from neighbouring sites
*wR*(*F*^2^) = 0.114	H-atom parameters constrained
*S* = 1.03	*w* = 1/[σ^2^(*F*_o_^2^) + (0.0516*P*)^2^ + 0.397*P*] where *P* = (*F*_o_^2^ + 2*F*_c_^2^)/3
6922 reflections	(Δ/σ)_max_ = 0.001
348 parameters	Δρ_max_ = 0.39 e Å^−^^3^
116 restraints	Δρ_min_ = −0.41 e Å^−^^3^

### Special details

**Table:**

Geometry. All esds (except the esd in the dihedral angle between two l.s. planes) are estimated using the full covariance matrix. The cell esds are taken into account individually in the estimation of esds in distances, angles and torsion angles; correlations between esds in cell parameters are only used when they are defined by crystal symmetry. An approximate (isotropic) treatment of cell esds is used for estimating esds involving l.s. planes.

### Fractional atomic coordinates and isotropic or equivalent isotropic displacement parameters (Å^2^)

**Table:**

	*x*	*y*	*z*	*U*_iso_*/*U*_eq_	Occ. (<1)
Sn1	0.43681 (3)	0.57720 (3)	0.210995 (12)	0.05681 (11)	
Cl1	0.26514 (16)	0.91485 (13)	0.09610 (6)	0.0829 (3)	
Cl2	0.86070 (17)	0.2126 (2)	0.18407 (7)	0.1150 (5)	
Cl3	0.2567 (3)	0.26908 (19)	0.23276 (8)	0.1215 (5)	
C1	0.1641 (5)	0.7088 (5)	0.19945 (18)	0.0605 (10)	
H1A	0.1026	0.6603	0.2294	0.073*	
H1B	0.1341	0.8167	0.2093	0.073*	
C2	0.1068 (3)	0.7144 (3)	0.13357 (9)	0.0509 (8)	
C3	0.0112 (3)	0.6280 (3)	0.12270 (11)	0.0618 (10)	
H3	−0.0205	0.5691	0.1562	0.074*	
C4	−0.0369 (3)	0.6296 (3)	0.06181 (13)	0.0714 (11)	
H4	−0.1008	0.5717	0.0545	0.086*	
C5	0.0105 (4)	0.7176 (3)	0.01178 (9)	0.0733 (12)	
H5	−0.0216	0.7187	−0.0290	0.088*	
C6	0.1061 (3)	0.8041 (3)	0.02265 (10)	0.0659 (10)	
H6	0.1378	0.8630	−0.0108	0.079*	
C7	0.1542 (3)	0.8025 (3)	0.08354 (11)	0.0549 (9)	
C8	0.5728 (5)	0.5071 (5)	0.1235 (2)	0.0676 (11)	
H8A	0.5150	0.5857	0.0891	0.081*	
H8B	0.6878	0.5044	0.1251	0.081*	
C9	0.5835 (3)	0.3466 (3)	0.11058 (13)	0.0559 (9)	
C10	0.4632 (3)	0.3387 (3)	0.07197 (13)	0.0745 (12)	
H10	0.3806	0.4317	0.0533	0.089*	
C11	0.4664 (4)	0.1919 (4)	0.06124 (15)	0.0962 (16)	
H11	0.3859	0.1866	0.0354	0.115*	
C12	0.5899 (5)	0.0529 (3)	0.08913 (18)	0.1005 (18)	
H12	0.5920	−0.0454	0.0820	0.121*	
C13	0.7102 (4)	0.0607 (3)	0.12775 (16)	0.0869 (15)	
H13	0.7928	−0.0323	0.1464	0.104*	
C14	0.7070 (3)	0.2076 (3)	0.13847 (12)	0.0664 (10)	
C15	0.4920 (6)	0.3911 (5)	0.2881 (2)	0.0772 (12)	
H15A	0.5436	0.2885	0.2718	0.093*	
H15B	0.5733	0.3995	0.3155	0.093*	
C16	0.3305 (4)	0.4030 (4)	0.32513 (14)	0.0729 (12)	
C17	0.2955 (5)	0.4740 (4)	0.38092 (16)	0.0969 (16)	
H17	0.3744	0.5073	0.3959	0.116*	
C18	0.1425 (6)	0.4953 (5)	0.41438 (15)	0.132 (3)	
H18	0.1191	0.5428	0.4517	0.159*	
C19	0.0246 (4)	0.4456 (5)	0.3920 (2)	0.136 (3)	
H19	−0.0778	0.4598	0.4144	0.164*	
C20	0.0596 (4)	0.3746 (5)	0.3362 (2)	0.122 (2)	
H20	−0.0193	0.3413	0.3213	0.146*	
C21	0.2126 (5)	0.3533 (4)	0.30278 (15)	0.0834 (13)	
O1	0.4575 (9)	0.7580 (9)	0.2569 (3)	0.0768 (14)	0.50
O2	0.7217 (9)	0.6062 (9)	0.2436 (3)	0.0887 (15)	0.50
O3	0.588 (4)	0.655 (3)	0.4097 (15)	0.104 (4)	0.50
O4	0.0927 (11)	0.9380 (10)	0.6204 (4)	0.1084 (18)	0.50
C22	0.6118 (11)	0.7264 (9)	0.2664 (3)	0.081 (2)	0.50
C23	0.6705 (14)	0.8200 (15)	0.3018 (6)	0.075 (3)	0.50
H23A	0.7752	0.7495	0.3227	0.090*	0.50
H23B	0.6982	0.8962	0.2722	0.090*	0.50
C24	0.548 (2)	0.9058 (18)	0.3491 (7)	0.071 (2)	0.50
H24A	0.4394	0.9684	0.3292	0.086*	0.50
H24B	0.5890	0.9788	0.3645	0.086*	0.50
C25	0.5198 (18)	0.802 (3)	0.4030 (6)	0.0772 (15)	0.50
C26	0.403 (2)	0.8541 (13)	0.4597 (6)	0.0708 (14)	0.50
C27	0.4017 (15)	0.7283 (9)	0.5032 (5)	0.081 (2)	0.50
H27	0.4724	0.6245	0.4964	0.097*	0.50
C28	0.2951 (11)	0.7578 (7)	0.5567 (4)	0.082 (2)	0.50
H28	0.2945	0.6736	0.5858	0.099*	0.50
C29	0.1895 (9)	0.9130 (8)	0.5668 (3)	0.072 (2)	0.50
C30	0.1905 (12)	1.0387 (7)	0.5234 (4)	0.081 (2)	0.50
H30	0.1198	1.1426	0.5301	0.097*	0.50
C31	0.2971 (18)	1.0093 (11)	0.4698 (5)	0.078 (2)	0.50
H31	0.2978	1.0934	0.4408	0.094*	0.50
C32	−0.022 (3)	1.0886 (15)	0.6320 (10)	0.124 (4)	0.50
H32A	−0.0767	1.0831	0.6727	0.187*	0.50
H32B	−0.1072	1.1294	0.6001	0.187*	0.50
H32C	0.0383	1.1578	0.6311	0.187*	0.50
O1'	0.3878 (8)	0.7681 (8)	0.2923 (3)	0.0768 (14)	0.50
O2'	0.5929 (9)	0.7007 (9)	0.2224 (3)	0.0887 (15)	0.50
O3'	0.611 (4)	0.677 (3)	0.3979 (15)	0.104 (4)	0.50
O4'	0.0474 (11)	1.0989 (9)	0.5980 (4)	0.1084 (18)	0.50
C22'	0.5231 (10)	0.7763 (8)	0.2700 (3)	0.081 (2)	0.50
C23'	0.6080 (14)	0.8686 (15)	0.2958 (6)	0.075 (3)	0.50
H23C	0.6144	0.9514	0.2640	0.090*	0.50
H23D	0.7237	0.7991	0.3079	0.090*	0.50
C24'	0.506 (2)	0.9411 (19)	0.3522 (7)	0.071 (2)	0.50
H24C	0.5528	1.0112	0.3666	0.086*	0.50
H24D	0.3883	1.0038	0.3407	0.086*	0.50
C25'	0.5114 (19)	0.816 (3)	0.4034 (6)	0.0772 (15)	0.50
C26'	0.389 (2)	0.8780 (13)	0.4571 (6)	0.0708 (14)	0.50
C27'	0.3492 (15)	0.7807 (8)	0.5055 (5)	0.081 (2)	0.50
H27'	0.3991	0.6709	0.5055	0.097*	0.50
C28'	0.2352 (11)	0.8477 (8)	0.5540 (3)	0.082 (2)	0.50
H28'	0.2088	0.7827	0.5864	0.099*	0.50
C29'	0.1607 (9)	1.0120 (8)	0.5540 (3)	0.072 (2)	0.50
C30'	0.2002 (12)	1.1092 (8)	0.5055 (4)	0.081 (2)	0.50
H30'	0.1504	1.2191	0.5055	0.097*	0.50
C31'	0.3142 (18)	1.0422 (12)	0.4571 (5)	0.078 (2)	0.50
H31'	0.3406	1.1073	0.4247	0.094*	0.50
C32'	−0.004 (3)	1.017 (2)	0.6466 (9)	0.124 (4)	0.50
H32D	0.0576	1.0085	0.6836	0.187*	0.50
H32E	0.0174	0.9126	0.6359	0.187*	0.50
H32F	−0.1249	1.0719	0.6549	0.187*	0.50

### Atomic displacement parameters (Å^2^)

**Table:**

	*U*^11^	*U*^22^	*U*^33^	*U*^12^	*U*^13^	*U*^23^
Sn1	0.05296 (17)	0.06063 (18)	0.05656 (17)	−0.02085 (13)	0.00286 (11)	−0.01208 (12)
Cl1	0.0876 (8)	0.0649 (6)	0.1080 (9)	−0.0445 (6)	−0.0020 (7)	−0.0017 (6)
Cl2	0.0662 (8)	0.1523 (14)	0.0965 (10)	−0.0022 (8)	−0.0191 (7)	−0.0315 (9)
Cl3	0.1578 (16)	0.1071 (11)	0.1053 (11)	−0.0565 (11)	−0.0304 (10)	0.0005 (9)
C1	0.057 (2)	0.058 (2)	0.059 (2)	−0.0125 (18)	0.0048 (18)	−0.0129 (18)
C2	0.0423 (19)	0.0439 (18)	0.059 (2)	−0.0094 (15)	0.0026 (16)	−0.0052 (16)
C3	0.054 (2)	0.059 (2)	0.071 (3)	−0.0237 (19)	0.0071 (19)	−0.0021 (19)
C4	0.067 (3)	0.076 (3)	0.081 (3)	−0.035 (2)	−0.005 (2)	−0.017 (2)
C5	0.071 (3)	0.078 (3)	0.061 (3)	−0.016 (2)	−0.013 (2)	−0.012 (2)
C6	0.064 (3)	0.057 (2)	0.064 (3)	−0.014 (2)	0.004 (2)	0.0029 (19)
C7	0.050 (2)	0.0436 (19)	0.067 (2)	−0.0148 (16)	0.0027 (18)	−0.0048 (17)
C8	0.063 (3)	0.071 (3)	0.067 (3)	−0.025 (2)	0.015 (2)	−0.011 (2)
C9	0.046 (2)	0.064 (2)	0.053 (2)	−0.0173 (18)	0.0114 (16)	−0.0103 (18)
C10	0.064 (3)	0.084 (3)	0.073 (3)	−0.027 (2)	0.007 (2)	−0.016 (2)
C11	0.072 (3)	0.123 (5)	0.109 (4)	−0.049 (3)	0.013 (3)	−0.043 (4)
C12	0.094 (4)	0.093 (4)	0.131 (5)	−0.054 (3)	0.045 (4)	−0.041 (4)
C13	0.077 (3)	0.064 (3)	0.096 (4)	−0.010 (2)	0.034 (3)	−0.002 (3)
C14	0.051 (2)	0.071 (3)	0.062 (2)	−0.009 (2)	0.0118 (18)	−0.009 (2)
C15	0.066 (3)	0.079 (3)	0.071 (3)	−0.014 (2)	−0.001 (2)	0.002 (2)
C16	0.072 (3)	0.061 (3)	0.068 (3)	−0.012 (2)	0.006 (2)	0.009 (2)
C17	0.107 (4)	0.090 (4)	0.075 (3)	−0.023 (3)	0.018 (3)	−0.006 (3)
C18	0.141 (6)	0.104 (5)	0.112 (5)	−0.016 (4)	0.051 (5)	−0.009 (4)
C19	0.098 (5)	0.125 (6)	0.151 (7)	−0.022 (4)	0.055 (5)	0.004 (5)
C20	0.087 (4)	0.109 (5)	0.159 (6)	−0.044 (4)	0.003 (4)	0.040 (4)
C21	0.085 (3)	0.070 (3)	0.087 (3)	−0.028 (3)	−0.002 (3)	0.015 (2)
O1	0.067 (3)	0.091 (3)	0.085 (4)	−0.038 (3)	0.004 (2)	−0.030 (3)
O2	0.088 (4)	0.109 (4)	0.082 (3)	−0.047 (3)	0.008 (3)	−0.034 (3)
O3	0.102 (6)	0.073 (5)	0.103 (9)	−0.007 (4)	0.016 (5)	0.012 (5)
O4	0.123 (5)	0.105 (4)	0.096 (4)	−0.042 (4)	0.001 (3)	−0.019 (3)
C22	0.087 (7)	0.088 (5)	0.076 (4)	−0.041 (5)	−0.008 (5)	−0.011 (4)
C23	0.070 (7)	0.090 (6)	0.078 (4)	−0.040 (6)	−0.010 (4)	−0.017 (4)
C24	0.075 (7)	0.065 (6)	0.077 (3)	−0.027 (5)	−0.007 (4)	−0.013 (3)
C25	0.073 (3)	0.072 (4)	0.078 (3)	−0.018 (3)	−0.016 (2)	−0.006 (2)
C26	0.066 (3)	0.073 (4)	0.070 (3)	−0.020 (3)	−0.019 (2)	−0.006 (3)
C27	0.082 (7)	0.077 (6)	0.074 (3)	−0.017 (5)	−0.009 (4)	−0.015 (4)
C28	0.084 (6)	0.074 (6)	0.079 (4)	−0.019 (5)	−0.018 (4)	−0.003 (5)
C29	0.070 (4)	0.066 (5)	0.080 (4)	−0.018 (4)	−0.012 (3)	−0.027 (4)
C30	0.082 (4)	0.072 (6)	0.086 (6)	−0.027 (4)	−0.007 (4)	−0.006 (5)
C31	0.076 (4)	0.078 (5)	0.077 (5)	−0.024 (3)	−0.011 (3)	−0.006 (4)
C32	0.117 (6)	0.163 (10)	0.105 (8)	−0.066 (8)	−0.002 (5)	−0.021 (7)
O1'	0.067 (3)	0.091 (3)	0.085 (4)	−0.038 (3)	0.004 (2)	−0.030 (3)
O2'	0.088 (4)	0.109 (4)	0.082 (3)	−0.047 (3)	0.008 (3)	−0.034 (3)
O3'	0.102 (6)	0.073 (5)	0.103 (9)	−0.007 (4)	0.016 (5)	0.012 (5)
O4'	0.123 (5)	0.105 (4)	0.096 (4)	−0.042 (4)	0.001 (3)	−0.019 (3)
C22'	0.087 (7)	0.088 (5)	0.076 (4)	−0.041 (5)	−0.008 (5)	−0.011 (4)
C23'	0.070 (7)	0.090 (6)	0.078 (4)	−0.040 (6)	−0.010 (4)	−0.017 (4)
C24'	0.075 (7)	0.065 (6)	0.077 (3)	−0.027 (5)	−0.007 (4)	−0.013 (3)
C25'	0.073 (3)	0.072 (4)	0.078 (3)	−0.018 (3)	−0.016 (2)	−0.006 (2)
C26'	0.066 (3)	0.073 (4)	0.070 (3)	−0.020 (3)	−0.019 (2)	−0.006 (3)
C27'	0.082 (7)	0.077 (6)	0.074 (3)	−0.017 (5)	−0.009 (4)	−0.015 (4)
C28'	0.084 (6)	0.074 (6)	0.079 (4)	−0.019 (5)	−0.018 (4)	−0.003 (5)
C29'	0.070 (4)	0.066 (5)	0.080 (4)	−0.018 (4)	−0.012 (3)	−0.027 (4)
C30'	0.082 (4)	0.072 (6)	0.086 (6)	−0.027 (4)	−0.007 (4)	−0.006 (5)
C31'	0.076 (4)	0.078 (5)	0.077 (5)	−0.024 (3)	−0.011 (3)	−0.006 (4)
C32'	0.117 (6)	0.163 (10)	0.105 (8)	−0.066 (8)	−0.002 (5)	−0.021 (7)

### Geometric parameters (Å, °)

**Table:**

Sn1—O2'	2.103 (7)	O3—C25	1.236 (9)
Sn1—O1	2.108 (7)	O4—C29	1.348 (7)
Sn1—C15	2.150 (4)	O4—C32	1.385 (9)
Sn1—C8	2.155 (4)	C22—C23	1.460 (9)
Sn1—C1	2.165 (4)	C23—C24	1.457 (8)
Sn1—O1'	2.527 (6)	C23—H23A	0.9700
Cl1—C7	1.706 (2)	C23—H23B	0.9700
Cl2—C14	1.705 (3)	C24—C25	1.469 (9)
Cl3—C21	1.722 (3)	C24—H24A	0.9700
C1—C2	1.513 (4)	C24—H24B	0.9700
C1—H1A	0.9700	C25—C26	1.510 (8)
C1—H1B	0.9700	C26—C27	1.3900
C2—C3	1.3900	C26—C31	1.3900
C2—C7	1.3900	C27—C28	1.3900
C3—C4	1.3900	C27—H27	0.9300
C3—H3	0.9300	C28—C29	1.3900
C4—C5	1.3900	C28—H28	0.9300
C4—H4	0.9300	C29—C30	1.3900
C5—C6	1.3900	C30—C31	1.3900
C5—H5	0.9300	C30—H30	0.9300
C6—C7	1.3900	C31—H31	0.9300
C6—H6	0.9300	C32—H32A	0.9600
C8—C9	1.505 (4)	C32—H32B	0.9600
C8—H8A	0.9700	C32—H32C	0.9600
C8—H8B	0.9700	O1'—C22'	1.237 (8)
C9—C10	1.3900	O2'—C22'	1.279 (8)
C9—C14	1.3900	O3'—C25'	1.240 (9)
C10—C11	1.3900	O4'—C32'	1.354 (9)
C10—H10	0.9300	O4'—C29'	1.379 (7)
C11—C12	1.3900	C22'—C23'	1.487 (9)
C11—H11	0.9300	C23'—C24'	1.496 (8)
C12—C13	1.3900	C23'—H23C	0.9700
C12—H12	0.9300	C23'—H23D	0.9700
C13—C14	1.3900	C24'—C25'	1.475 (9)
C13—H13	0.9300	C24'—H24C	0.9700
C15—C16	1.504 (5)	C24'—H24D	0.9700
C15—H15A	0.9700	C25'—C26'	1.499 (8)
C15—H15B	0.9700	C26'—C27'	1.3900
C16—C17	1.3900	C26'—C31'	1.3900
C16—C21	1.3900	C27'—C28'	1.3900
C17—C18	1.3900	C27'—H27'	0.9300
C17—H17	0.9300	C28'—C29'	1.3900
C18—C19	1.3900	C28'—H28'	0.9300
C18—H18	0.9300	C29'—C30'	1.3900
C19—C20	1.3900	C30'—C31'	1.3900
C19—H19	0.9300	C30'—H30'	0.9300
C20—C21	1.3900	C31'—H31'	0.9300
C20—H20	0.9300	C32'—H32D	0.9600
O1—C22	1.251 (8)	C32'—H32E	0.9600
O2—C22	1.260 (8)	C32'—H32F	0.9600
			
O2'—Sn1—O1	35.4 (2)	C20—C21—Cl3	120.8 (3)
O2'—Sn1—C15	106.1 (2)	C16—C21—Cl3	119.2 (3)
O1—Sn1—C15	99.8 (2)	C22—O1—Sn1	109.7 (6)
O2'—Sn1—C8	89.2 (2)	C29—O4—C32	121.6 (11)
O1—Sn1—C8	122.1 (2)	O1—C22—O2	117.3 (8)
C15—Sn1—C8	115.29 (17)	O1—C22—C23	124.0 (8)
O2'—Sn1—C1	119.2 (2)	O2—C22—C23	118.7 (8)
O1—Sn1—C1	91.8 (2)	C24—C23—C22	114.9 (8)
C15—Sn1—C1	112.27 (17)	C24—C23—H23A	108.5
C8—Sn1—C1	112.93 (16)	C22—C23—H23A	108.5
O2'—Sn1—O1'	54.5 (2)	C24—C23—H23B	108.5
O1—Sn1—O1'	20.3 (2)	C22—C23—H23B	108.5
C15—Sn1—O1'	87.3 (2)	H23A—C23—H23B	107.5
C8—Sn1—O1'	142.43 (18)	C23—C24—C25	113.6 (8)
C1—Sn1—O1'	81.82 (17)	C23—C24—H24A	108.8
C2—C1—Sn1	113.2 (2)	C25—C24—H24A	108.8
C2—C1—H1A	108.9	C23—C24—H24B	108.8
Sn1—C1—H1A	108.9	C25—C24—H24B	108.8
C2—C1—H1B	108.9	H24A—C24—H24B	107.7
Sn1—C1—H1B	108.9	O3—C25—C24	125.8 (14)
H1A—C1—H1B	107.7	O3—C25—C26	107.8 (18)
C3—C2—C7	120.0	C24—C25—C26	126.4 (15)
C3—C2—C1	120.1 (2)	C27—C26—C31	120.0
C7—C2—C1	119.8 (2)	C27—C26—C25	113.4 (11)
C4—C3—C2	120.0	C31—C26—C25	126.5 (11)
C4—C3—H3	120.0	C26—C27—C28	120.0
C2—C3—H3	120.0	C26—C27—H27	120.0
C3—C4—C5	120.0	C28—C27—H27	120.0
C3—C4—H4	120.0	C29—C28—C27	120.0
C5—C4—H4	120.0	C29—C28—H28	120.0
C4—C5—C6	120.0	C27—C28—H28	120.0
C4—C5—H5	120.0	O4—C29—C28	118.5 (6)
C6—C5—H5	120.0	O4—C29—C30	121.4 (6)
C7—C6—C5	120.0	C28—C29—C30	120.0
C7—C6—H6	120.0	C29—C30—C31	120.0
C5—C6—H6	120.0	C29—C30—H30	120.0
C6—C7—C2	120.0	C31—C30—H30	120.0
C6—C7—Cl1	119.14 (15)	C30—C31—C26	120.0
C2—C7—Cl1	120.82 (15)	C30—C31—H31	120.0
C9—C8—Sn1	112.1 (2)	C26—C31—H31	120.0
C9—C8—H8A	109.2	C22'—O1'—Sn1	84.2 (5)
Sn1—C8—H8A	109.2	C22'—O2'—Sn1	103.2 (6)
C9—C8—H8B	109.2	C32'—O4'—C29'	117.2 (11)
Sn1—C8—H8B	109.2	O1'—C22'—O2'	117.8 (8)
H8A—C8—H8B	107.9	O1'—C22'—C23'	123.4 (8)
C10—C9—C14	120.0	O2'—C22'—C23'	118.8 (8)
C10—C9—C8	118.9 (3)	C22'—C23'—C24'	108.8 (7)
C14—C9—C8	121.1 (3)	C22'—C23'—H23C	109.9
C9—C10—C11	120.0	C24'—C23'—H23C	109.9
C9—C10—H10	120.0	C22'—C23'—H23D	109.9
C11—C10—H10	120.0	C24'—C23'—H23D	109.9
C12—C11—C10	120.0	H23C—C23'—H23D	108.3
C12—C11—H11	120.0	C25'—C24'—C23'	110.2 (8)
C10—C11—H11	120.0	C25'—C24'—H24C	109.6
C11—C12—C13	120.0	C23'—C24'—H24C	109.6
C11—C12—H12	120.0	C25'—C24'—H24D	109.6
C13—C12—H12	120.0	C23'—C24'—H24D	109.6
C12—C13—C14	120.0	H24C—C24'—H24D	108.1
C12—C13—H13	120.0	O3'—C25'—C24'	118.9 (14)
C14—C13—H13	120.0	O3'—C25'—C26'	128.2 (19)
C13—C14—C9	120.0	C24'—C25'—C26'	112.9 (15)
C13—C14—Cl2	118.7 (2)	C27'—C26'—C31'	120.0
C9—C14—Cl2	121.2 (2)	C27'—C26'—C25'	123.5 (11)
C16—C15—Sn1	110.4 (3)	C31'—C26'—C25'	116.5 (11)
C16—C15—H15A	109.6	C28'—C27'—C26'	120.0
Sn1—C15—H15A	109.6	C28'—C27'—H27'	120.0
C16—C15—H15B	109.6	C26'—C27'—H27'	120.0
Sn1—C15—H15B	109.6	C27'—C28'—C29'	120.0
H15A—C15—H15B	108.1	C27'—C28'—H28'	120.0
C17—C16—C21	120.0	C29'—C28'—H28'	120.0
C17—C16—C15	118.6 (3)	O4'—C29'—C28'	128.1 (6)
C21—C16—C15	121.3 (3)	O4'—C29'—C30'	111.9 (6)
C18—C17—C16	120.0	C28'—C29'—C30'	120.0
C18—C17—H17	120.0	C31'—C30'—C29'	120.0
C16—C17—H17	120.0	C31'—C30'—H30'	120.0
C17—C18—C19	120.0	C29'—C30'—H30'	120.0
C17—C18—H18	120.0	C30'—C31'—C26'	120.0
C19—C18—H18	120.0	C30'—C31'—H31'	120.0
C18—C19—C20	120.0	C26'—C31'—H31'	120.0
C18—C19—H19	120.0	O4'—C32'—H32D	109.5
C20—C19—H19	120.0	O4'—C32'—H32E	109.5
C21—C20—C19	120.0	H32D—C32'—H32E	109.5
C21—C20—H20	120.0	O4'—C32'—H32F	109.5
C19—C20—H20	120.0	H32D—C32'—H32F	109.5
C20—C21—C16	120.0	H32E—C32'—H32F	109.5
			
O2'—Sn1—C1—C2	109.8 (3)	Sn1—O1—C22—O2	5.3 (5)
O1—Sn1—C1—C2	133.3 (3)	Sn1—O1—C22—C23	−174.8 (5)
C15—Sn1—C1—C2	−125.3 (3)	O1—C22—C23—C24	26.9 (10)
C8—Sn1—C1—C2	7.1 (3)	O2—C22—C23—C24	−153.2 (10)
O1'—Sn1—C1—C2	151.1 (3)	C22—C23—C24—C25	68.8 (14)
Sn1—C1—C2—C3	108.5 (2)	C23—C24—C25—O3	−0.9 (14)
Sn1—C1—C2—C7	−69.5 (3)	C23—C24—C25—C26	179.3 (14)
C7—C2—C3—C4	0.0	O3—C25—C26—C27	−0.1 (11)
C1—C2—C3—C4	−178.1 (3)	C24—C25—C26—C27	179.8 (11)
C2—C3—C4—C5	0.0	O3—C25—C26—C31	−177.5 (17)
C3—C4—C5—C6	0.0	C24—C25—C26—C31	2.4 (17)
C4—C5—C6—C7	0.0	C31—C26—C27—C28	0.0
C5—C6—C7—C2	0.0	C25—C26—C27—C28	−177.6 (13)
C5—C6—C7—Cl1	−177.78 (19)	C26—C27—C28—C29	0.0
C3—C2—C7—C6	0.0	C32—O4—C29—C28	−176.6 (14)
C1—C2—C7—C6	178.1 (3)	C32—O4—C29—C30	6.1 (17)
C3—C2—C7—Cl1	177.74 (19)	C27—C28—C29—O4	−177.4 (10)
C1—C2—C7—Cl1	−4.2 (3)	C27—C28—C29—C30	0.0
O2'—Sn1—C8—C9	148.1 (3)	O4—C29—C30—C31	177.3 (10)
O1—Sn1—C8—C9	161.9 (3)	C28—C29—C30—C31	0.0
C15—Sn1—C8—C9	40.7 (3)	C29—C30—C31—C26	0.0
C1—Sn1—C8—C9	−90.2 (3)	C27—C26—C31—C30	0.0
O1'—Sn1—C8—C9	162.5 (3)	C25—C26—C31—C30	177.2 (15)
Sn1—C8—C9—C10	96.0 (3)	O2'—Sn1—O1'—C22'	−2.6 (3)
Sn1—C8—C9—C14	−81.6 (3)	O1—Sn1—O1'—C22'	−18.9 (7)
C14—C9—C10—C11	0.0	C15—Sn1—O1'—C22'	109.4 (3)
C8—C9—C10—C11	−177.6 (3)	C8—Sn1—O1'—C22'	−20.4 (5)
C9—C10—C11—C12	0.0	C1—Sn1—O1'—C22'	−137.7 (3)
C10—C11—C12—C13	0.0	O1—Sn1—O2'—C22'	12.3 (4)
C11—C12—C13—C14	0.0	C15—Sn1—O2'—C22'	−72.0 (4)
C12—C13—C14—C9	0.0	C8—Sn1—O2'—C22'	171.8 (4)
C12—C13—C14—Cl2	−177.9 (2)	C1—Sn1—O2'—C22'	55.8 (5)
C10—C9—C14—C13	0.0	O1'—Sn1—O2'—C22'	2.6 (3)
C8—C9—C14—C13	177.5 (3)	Sn1—O1'—C22'—O2'	3.9 (4)
C10—C9—C14—Cl2	177.9 (2)	Sn1—O1'—C22'—C23'	−175.9 (4)
C8—C9—C14—Cl2	−4.6 (3)	Sn1—O2'—C22'—O1'	−4.8 (5)
O2'—Sn1—C15—C16	123.7 (3)	Sn1—O2'—C22'—C23'	175.1 (5)
O1—Sn1—C15—C16	87.9 (4)	O1'—C22'—C23'—C24'	1.8 (9)
C8—Sn1—C15—C16	−139.4 (3)	O2'—C22'—C23'—C24'	−178.1 (9)
C1—Sn1—C15—C16	−8.1 (4)	C22'—C23'—C24'—C25'	65.6 (13)
O1'—Sn1—C15—C16	71.9 (3)	C23'—C24'—C25'—O3'	9.6 (14)
Sn1—C15—C16—C17	−99.6 (3)	C23'—C24'—C25'—C26'	−170.3 (14)
Sn1—C15—C16—C21	76.6 (3)	O3'—C25'—C26'—C27'	−11.1 (12)
C21—C16—C17—C18	0.0	C24'—C25'—C26'—C27'	168.8 (12)
C15—C16—C17—C18	176.2 (3)	O3'—C25'—C26'—C31'	168.3 (14)
C16—C17—C18—C19	0.0	C24'—C25'—C26'—C31'	−11.8 (14)
C17—C18—C19—C20	0.0	C31'—C26'—C27'—C28'	0.0
C18—C19—C20—C21	0.0	C25'—C26'—C27'—C28'	179.4 (15)
C19—C20—C21—C16	0.0	C26'—C27'—C28'—C29'	0.0
C19—C20—C21—Cl3	−178.7 (3)	C32'—O4'—C29'—C28'	−2.5 (17)
C17—C16—C21—C20	0.0	C32'—O4'—C29'—C30'	178.2 (14)
C15—C16—C21—C20	−176.1 (3)	C27'—C28'—C29'—O4'	−179.2 (11)
C17—C16—C21—Cl3	178.8 (3)	C27'—C28'—C29'—C30'	0.0
C15—C16—C21—Cl3	2.6 (3)	O4'—C29'—C30'—C31'	179.3 (9)
O2'—Sn1—O1—C22	−30.9 (4)	C28'—C29'—C30'—C31'	0.0
C15—Sn1—O1—C22	73.1 (4)	C29'—C30'—C31'—C26'	0.0
C8—Sn1—O1—C22	−55.2 (5)	C27'—C26'—C31'—C30'	0.0
C1—Sn1—O1—C22	−173.9 (4)	C25'—C26'—C31'—C30'	−179.4 (14)
O1'—Sn1—O1—C22	125.8 (10)		
